# Protective Efficacy of Coccidial Common Antigen Glyceraldehyde 3-Phosphate Dehydrogenase (GAPDH) against Challenge with Three *Eimeria* Species

**DOI:** 10.3389/fmicb.2017.01245

**Published:** 2017-07-18

**Authors:** Lu Tian, Wenyu Li, Xinmei Huang, Di Tian, Jianhua Liu, Xinchao Yang, Lianrui Liu, Ruofeng Yan, Lixin Xu, Xiangrui Li, Xiaokai Song

**Affiliations:** ^1^College of Veterinary Medicine, Nanjing Agricultural University Nanjing, China; ^2^Institute of Veterinary Medicine, Jiangsu Academy of Agricultural Sciences Nanjing, China

**Keywords:** chicken coccidian, common antigen, GAPDH, mixed infection, immunogenicity, protection

## Abstract

Coccidiosis is an intestinal disorder of poultry and often caused by simultaneous infections of several *Eimeria* species. GAPDH is one of the immunogenic common antigens among *Eimeria tenella*, *E. acervulina*, and *E. maxima* identified in our previous study. The present study was performed to further evaluate its immunogenicity and protective efficacy. The genes of *GAPDH* cloned from *E. acervulina* and *E. maxima* were named as *EaGAPDH* and *EmGAPDH*, respectively. The immunogenicity of recombinant proteins of EaGAPDH and EmGAPDH were analyzed by Western blot. The transcription and expression of pVAX-EaGAPDH and pVAX-EmGAPDH in the injected muscles were detected by reverse transcription PCR (RT-PCR) and Western blot, respectively. GAPDH-induced changes of T lymphocytes subpopulation, cytokines production, and antibody were determined using flow cytometry, quantitative real-time PCR (qPCR), and ELISA, respectively. Finally, the protective efficacies of pVAX-EaGAPDH and pVAX-EmGAPDH were evaluated by vaccination and challenge experiments. The results revealed that the recombinant GAPDH proteins reacted with the corresponding chicken antisera. The *EaGAPDH* genes were successfully transcribed and expressed in the injected muscles. Vaccination with pVAX-EaGAPDH and pVAX-EmGAPDH significantly increased the proportion of CD4^+^ and CD8^+^ T lymphocytes, the cytokines productions of IFN-γ, IL-2, IL-4 et al., and IgG antibody levels compared to controls. The vaccination increased the weight gains, decreased the oocyst outputs, alleviate the enteric lesions compared to controls, and induced moderate anti-coccidial index (ACI). In conclusion, the coccidial common antigen of GAPDH induced significant humoral and cellular immune response and effective protection against *E. tenella*, *E. acervulina*, *E. maxima*, and mixed infection of the three *Eimeria* species.

## Introduction

Chicken coccidiosis, the major parasitic disease of poultry, was caused by multiple *Eimeria* species (Carvalho et al., 2011; Ogedengbe et al., 2011; Kumar et al., 2014). Coccidiosis seriously impairs the growth and feed utilization of infected chickens resulting in loss of productivity and inflicts tremendous economic losses to the world poultry industry in excess of US$3 billion annually(Blake and Tomley, 2014; Witcombe and Smith, 2014). The species of *E. tenella*, *E. acervulina*, and *E. maxima* are the most important in terms of global disease burden and economic impact (Blake and Tomley, 2014; Reid et al., 2014). Conventional control strategies still rely heavily on chemoprophylaxis or live vaccines. However, the problems of drug residues, drug resistance, and the security and high cost of live vaccine direct our attentions to new generation vaccine, such as recombinant vaccine and DNA vaccine (Vermeulen, 1998; Clarke et al., 2014; Ahmad et al., 2016; Meunier et al., 2016). Under natural conditions, chicken coccidiosis is commonly caused by co-infections of several *Eimeria* species (Carvalho et al., 2011; Ogedengbe et al., 2011; del Cacho et al., 2012). Furthermore, protective immunity elicited by a given *Eimeria* species is species specific (Dalloul and Lillehoj, 2006). An ideal practical field vaccine should include common protective antigens among several *Eimeria* species and be able to induce effective protection against all the economically important species of *Eimeria* (del Cacho et al., 2012).

Some *Eimeria* common antigens have been reported in previous studies. Talebi (1995) only reported the size of the common immunogenic protein, Sasai et al. (1996) and Constantinoiu et al. (2003) reported that the common antigen was apical antigen. They did not identify the specific common antigens by sequencing. In addition, they did not evaluate the protective efficacies of the common antigens.

It is considered that humoral immunity plays minor role, and cell-mediated, especially Th1-type immunity plays major role in protective immunity against *Eimeria* infection (Dalloul and Lillehoj, 2006; Chapman, 2014). The Th1-type cytokines, such as IFN-γ and IL-2, are responsible for classic cell-mediated functions and seem to be dominant during coccidiosis (Lowenthal et al., 1997; Cornelissen et al., 2009). Hence, in this study, the proportion of CD4^+^ and CD8^+^ T lymphocytes, the Th1-type cytokines productions and IgG antibody levels were measured to evaluate humoral and cellular immune response induced by coccidial common antigen GAPDH.

In an initial screen, we identified five specific *Eimeria* common immunogenic antigens among sporozoites of *E. tenella*, *E. acervulina*, and *E. maxima* by immunoproteomic analysis (Liu et al., 2017). GAPDH, one of the five identified *Eimeria* common immunogenic antigens, is highly conserved among all chicken *Eimeria* species. GAPDH is a key glycolytic enzyme in the process of metabolism of coccidian, as several pathogenic protozoa entirely depend on glycolysis as the source of ATP in the host. Thus, protozoal GAPDHs are considered potential targets for anti-protozoan drugs (Bruno et al., 2016, 2017).

Here, we presented the extension work on *Eimeria* common antigen GAPDH identified in our previous study. We analyzed the immunogenicity of GAPDH and evaluated the protective efficacy of GAPDH against challenge with *E. tenella*, *E. maxima* and *E. acervulina*. Our data demonstrated that GAPDH could be selected as candidate antigen for the development of multivalent vaccine against co-infections of multiple *Eimeria* species in poultry farms.

## Materials and Methods

### Plasmids, Parasites, and Animals

**FIGURE 1:**
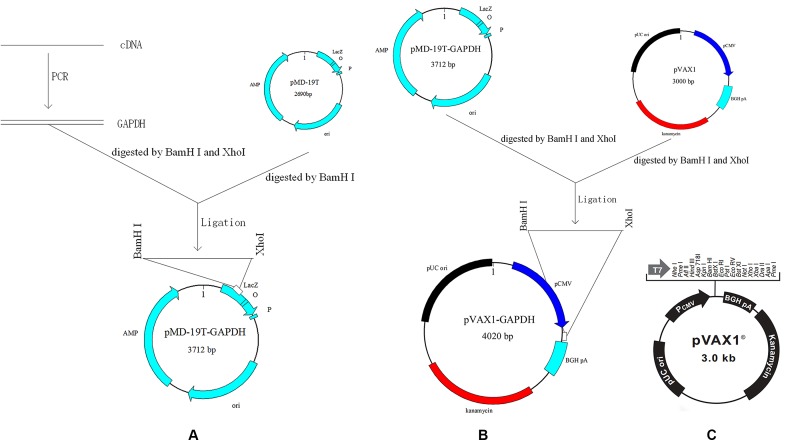
Scheme of cloning GAPDH into vectors. **(A)** Cloning GAPDH into pMD-19T. **(B)** Cloning GAPDH into pVAX1. **(C)** Map of eukaryotic expression vector pVAX1.

The prokaryotic expression vector pET-32a was purchased from Novagen (Darmstadt, Germany), and the eukaryotic expression vector pVAX1 (**Figure 1C**) was purchased from Invitrogen (Carlsbad, CA, United States). *E. tenella*, *E. acervuline*, and *E. maxima* were isolated from Jiangsu Province of China (JS). And their purity were determined with ITS1-PCR as described previously (Jenkins et al., 2006; Haug et al., 2007). Oocysts of *E. tenella*, *E. acervuline*, and *E. maxima* were propagated, harvested and sporulated using a previously described protocol 7 days prior to the challenge infection (Tomley, 1997). New-hatched Hy-Line layer chickens (commercial breed W-36) were raised in a sterilized room under coccidia-free conditions until the end of the experiment. Food and water without anti-coccidial drugs were available. Thirty-day-old rats (SD) were obtained from the Comparative Medicine Centre, Yangzhou University, Yangzhou, China. Animal experiments were conducted following the guidelines of the Animal Ethics Committee, Nanjing Agricultural University, China. All animal experiments were evaluated and approved by the Institutional Animal Care and Use Committee of Nanjing Agricultural University (approval number: 2012CB120750).

### Cloning of *Eimeria GAPDH* Genes

Sporulated oocysts of *E. acervulina* and *E. maxima* were broken to release sporocysts by whirl mix with micro glass balls (Tomley, 1997). Next, total RNA was extracted from the released sporocysts of *E. acervulina* and *E. maxima* using E.Z.N.A.^®^ Total RNA Kit Maxi Kit (OMEGA, Norcross, GA, United States), respectively. **Figure 1A** showed schematic of the cloning strategy. The cDNAs were synthesized by reverse transcription (RT) reaction with the specific primers for *E. acervulina GAPDH* (*EaGAPDH*) and *E. maxima GAPDH* (*EmGAPDH*) (see **Table 1**). PCR products were cloned into pMD-19T vector (TaKaRa, Dalian, China) to construct pMD-19T-EaGAPDH and pMD-19T-EmGAPDH. The recombinant plasmids were identified by endonuclease digestion and sequencing. The nucleotide sequences were analyzed online with a basic alignment search tool (BLAST)^1^.

**Table 1:** Primers for *Eimeria acervulina* GAPDH and *E. maxima* GAPDH.

Gene	Primer	Size (aa)
EaGAPDH	Forward: 5′-CGCGGATCCATGGTGTGCCGTATGGGAATCA-3′ Reverse: 5′-CCGCTCGAGTTAGTTGCCGTCCTTCTTAGACATGTAG-3′	340
EmGAPDH	Forward: 5′-CGCGGATCCATGGTTTGCCGCATGGGC-3′ Reverse: 5′-CCGCTCGAGTCAGTTGCCGTCCTTCTTGGAC -3′	340

*EaGAPDH = E. acervulina GAPDH; EmGAPDH = E. maxima GAPDH; aa = amino acids.*

### Preparation of GAPDH Recombinant Proteins and Anti-GAPDH Sera

The *EaGAPDH* and *EmGAPDH* genes were cloned into the prokaryotic expression vector pET-32a, and transformed into *E. coli* BL21 (DE3), respectively. The recombinant proteins were expressed and purified with His Trap^TM^ FF (GE Healthcare, United States) following introduction of the kit. The purities of protein were determined by SDS-PAGE and protein concentrations were estimated using Pierce^TM^ BCA Protein Assay Kit (Thermo Scientific, Waltham, MA, United States). The proteins were diluted in PBS buffer with a concentration of 400 μg/ml. Stocks proteins were prepared and stored at –80°C until further use.

Rat anti-EaGAPDH and anti-EmGAPDH sera were prepared as following. Rats were vaccinated subcutaneously in the back with 200 μg (400 μg/ml) recombinant proteins of EaGAPDH and EmGAPDH emulsified in 0.5 ml of Freund’s Adjuvant Complete (Sigma–Aldrich, Merck KGaA, Darmstadt, Germany) at 30 days of age separately. Two weeks later, two doses of proteins (200 μg, 400 μg/ml) emulsified in 0.5 ml of Freund’s Adjuvant Incomplete (Sigma–Aldrich) were given at an interval of 1 week. A forth even fifth dose would be given unless the titers of sera were beyond 1: 64. The antisera were collected and then stored at –20°C for further Western blot analysis.

### Western Blot Analysis of GAPDH Recombinant Proteins with Chicken Anti-*Eimeria* Sera

Chicken Antisera were obtained after two oral infections of chickens with sporulated oocysts of *E. acervulina* and *E. maxima* at 10-days intervals and bled 10 days after the last infection (Song et al., 2017). Serum was collected from the uninfected chicken as empty control. Recombinant proteins of EaGAPDH and EmGAPDH were analyzed by Western blot assay with anti-*E. acervulina* and anti-*E. maxima* chicken sera as first antibody, respectively. Briefly, recombinant proteins were separated by sodium dodecyl sulfate–polyacrylamide gel electrophoresis (SDS–PAGE) and then transferred to a nitrocellulose membrane (Bio-Rad, Hercules, CA, United States). The membrane was incubated with anti-*E. acervulina* and anti-*E. maxima* chicken sera (1: 100) and horseradish peroxidase (HRP)-conjugated Goat anti-chicken IgG (1:2000, Sigma–Aldrich) as secondary antibody. The bound antibody was detected using 3, 30-diaminobenzidine (DAB) (Song et al., 2015b).

### Construction of Recombinant Plasmids pVAX-EaGAPDH and pVAX-EmGAPDH

Recombinant plasmids pVAX-EaGAPDH and pVAX-EmGAPDH were generated with the eukaryotic expression vector pVAX1. The cloning scheme was shown in **Figure 1B**. Briefly, fragments of EaGAPDH and EmGAPDH were excised from the plasmids of pMD19-EaGAPDH and pMD19-EmGAPDH by BamHI and XhoI (TaKaRa) digestion and ligated into pVAX1 at the same enzyme sites to construct pVAX-EaGAPDH and pVAX-EmGAPDH, respectively. The resulting plasmids were confirmed by endonuclease cleavage and sequence analysis.

### Transcription and Expression Detection of the *GAPDH* Genes *In Vivo* by Reverse Transcription PCR and Western Blot

Chickens were intramuscularly injected with the recombinant plasmids pVAX-EaGAPDH and pVAX-EmGAPDH in thighs at 14 days of age separately. Seven days later, about 0.5 g of the injected muscle was cut for transcription and expression detection of the *GAPDH* genes. Meanwhile the same site of muscles from non-injected chickens was selected as control.

Total mRNA from the injected muscle was extracted and digested by DNase I (TaKaRa) to remove the residual plasmid. With the specific primers for *GAPDH* genes (**Table 1**), RT-PCR were performed with the product RNA as template and electrophoresis in 1% agarose gel was performed to examine the transcription of *EaGAPDH* and *EmGAPDH.*

The injected muscle was treated with RIPA solution [0.1 mol/L phenylmethylsulfonyl fluoride (PMSF), 50 mmol/L Tris–HCl, 150 mmol/L NaCl, 1% Nonnidet P-40, 0.1% SDS] for 3 h. After centrifugation at 13,000 rpm, the supernatant was collected for use. Then Western blots were performed to detect the expressed proteins with anti-EaGAPDH and anti-EmGAPDH rat sera as primary antibody.

### Immunogenicity Evaluation of GAPDH in Chickens

#### Immunization with Recombinant Plasmids of pVAX-EaGAPDH and pVAX-EmGAPDH

**FIGURE 2:**
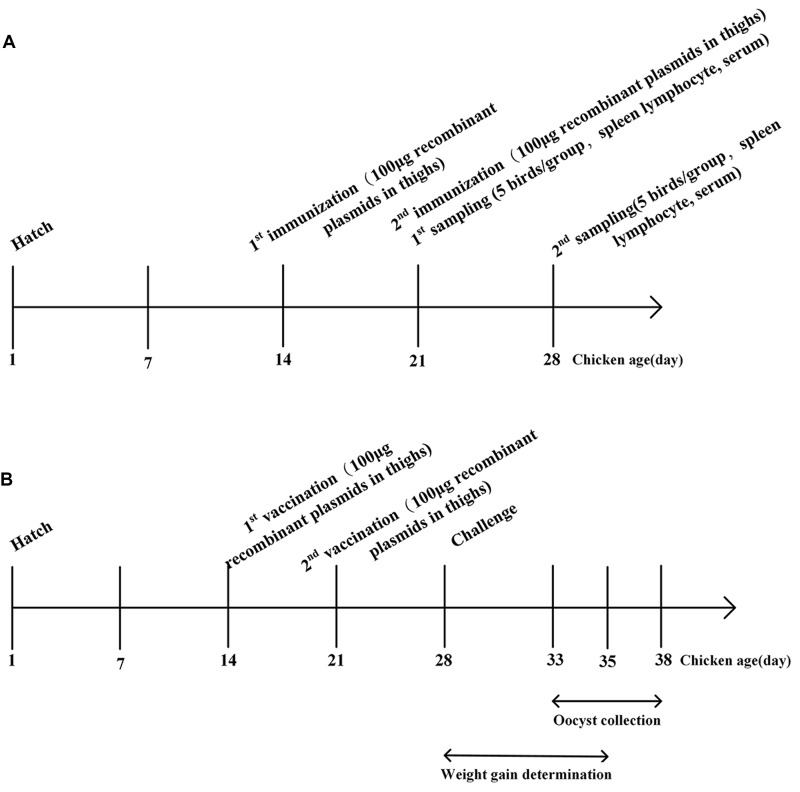
Schematic outlines of the experimental design. **(A)** Schematic outline for immunogenicity evaluation of GAPDH in chickens. **(B)** Schematic outline for protective efficacy evaluation of GAPDH.

Two-week-old chickens were randomly divided into four groups of 15 chickens each. The schematic of vaccination was shown in **Figure 2A**. Group 1 and 2 were intramuscularly vaccinated with 100 μg (0.5 μg/μl) of recombinant plasmids pVAX-EaGAPDH and pVAX-EmGAPDH in thighs, respectively. Group 3 was injected with 100 μg (0.5 μg/μl) of empty pVAX1. Group 4 was injected with sterile phosphate buffered saline (PBS). One week later, a booster injection was given with the same way as the primary vaccination. One week after the primary and booster vaccination, spleens were collected from five chickens of each group to evaluate the induced changes of T lymphocytes subpopulations and cytokines production separately. The rest five chickens from each group were used to collect blood sera for specific antibody determination.

### Determination of GAPDH-Induced Changes in T Lymphocyte Subpopulations by Flow Cytometry Analysis

The proportions of spleen T lymphocyte subpopulations of CD4+ and CD8+ from the vaccinated chickens were analyzed using flow cytometry technique. One week after the primary and booster vaccination, spleens were collected from five chickens of each group to prepare the spleen lymphocytes as described by Sasai et al. (2000). The spleens were cut into scraps and gently pushed through a mesh screen and cells were washed with calcium and magnesium-free Hank’s balanced salt solution (HBSS). Lymphocytes were collected using a separation solution (TBD, Tianjin, China) according to the manufacturer’s protocol. Lymphocytes suspensions (1 × 10^6^ cells/ml) were incubated with anti-chicken CD3-FITC, anti-chicken CD3-FITC + anti-chicken CD8-PE, anti-chicken CD3-FITC + anti-chicken CD4-PE (SouthernBiotech, Birmingham, AL, United States) in the dark for 30 min at 4°C separately. Subsequently, the cells were washed twice with PBS by centrifugation (500 ×*g*, 10 min, 4°C) and re-suspended with cold PBS and analyzed with a BD FACSCalibur flow cytometer (BD, Franklin Lakes, NJ, United States). A lymphocyte specific gating was set according to forward and side scatter profiles. The proportions of CD4+ and CD8+ T lymphocytes were determined as described by (Song et al., 2010).

### Determination of GAPDH-Induced Changes in Cytokines by Quantitative Real-Time PCR

The transcription levels of IFN-γ, IL-2, IL-4 TNFSF15, IL-17, and TGF-β4 were determined by qPCR. Total RNA was extracted from the isolated lymphocytes of spleen from chickens (five per group) using E.Z.N.A.^®^ Total RNA Kit Maxi Kit (OMEGA, Norcross, GA, United States). Lymphocytes cDNA was synthetized using HiScript II Q RT SuperMix (Vazyme, Nanjing, China). Chicken GAPDH gene was used as an internal control. The primers for qPCR were presented in **Table 2** (Song et al., 2010). Amplifications were conducted in 20 μl reaction mixture containing 1 μl of cDNA, 10 μl of 2 × ChamQ^TM^ SYBR^®^ qPCR Master Mix (Vazyme, Nanjing, China), and 0.5 μM primers. Reaction was performed in an ABI PRISM 7300 Fast Real-Time PCR System (Applied Biosystems, Carlsbad, CA, United States), with an initial denaturation at 95°C for 30 s, followed by 40 cycles at 95°C for 10 s, 60°C for 30 s and followed by a melting curve program at 95°C 15 s, 60°C 15 s, 95°C 15 s. Each reaction was analyzed in triplicate and GAPDH was used as endogenous control. A negative control, in which template cDNA was replaced by PCR-grade water was included with all qPCR assays. In this study, the 2^-ΔΔCT^ method was used to estimate the relative quantification of cytokine gene mRNA compared with the internal control gene of GAPDH (n-fold change to the water control group) (Livak and Schmittgen, 2001). Validation experiment was performed by running a dilution series of the cDNA to evaluate the amplification efficiencies of the target genes and reference genes (Song et al., 2010). The corresponding real-time efficiency (E) of one cycle in the exponential phase was calculated by the following formula:

**Table 2:** Primers used for the quantitative RT-PCR.

RNA target	Primer sequence	Accession No.	Amplification efficiency (%)^∗^	Correlation coefficients (*r*^2^)
IFN-γ	Forward: 5′-AGCTGACGGTGGACCTATTATT-3′	Y07922	99.16	0.9976
	Reverse: 5′-GGCTTTGCGCTGGATTC-3′			
IL-2	Forward: 5′-TCTGGGACCACTGTATGCTCT-3′	AF000631	98.53	0.9932
	Reverse: 5′-ACACCAGTGGGAAACAGTATCA-3′			
TNFSF15	Forward: 5′-CCTGAGTTATTCCAGCAACGCA-3′	NM_001024578	98.51	0.9992
	Reverse: 5′-ATCCACCAGCTTGATGTCACTAAC-3′			
IL-17D	Forward: 5′-GCTGCCTCATGGGGATCTTTGGTG-3′	EF570583	98.18	0.9954
	Reverse: 5′-CGATGACGGCTTGTTCTGGTTGAC-3′			
TGF-β4	Forward: 5′-CGGGACGGATGAGAAGAAC-3′	M31160	97.39	0.9981
	Reverse: 5′-CGGCCCACGTAGTAAATGAT-3′			
IL-4	Forward: 5′-ACCCAGGGCATCCAGAAG-3′	AJ621735	99.41	0.9996
	Reverse: 5′-CAGTGCCGGCAAGAAGTT-3′			
GAPDH	Forward: 5′-GGTGGTGCTAAGCGTGTTAT-3′	K01458	95.48	0.9994
	Reverse: 5′-ACCTCTGTCATCTCTCCACA-3′			

*^∗^Amplification efficiency (%) = (10^-1/slope^–1) × 100.*

$$E=10^{-1/slope}-1(Sanchezet\,\;al.,2006).$$

As the efficiency of the cytokine primers were not 100% (Song et al., 2010), Pfaffl correction was done for these RT-PCR analyses.

### Determination of GAPDH-Specific IgG Antibody Response by ELISA

After 1 week of primary and booster vaccination, whole blood from five chickens per group was collected from wing vein of the chickens and the sera were collected for determining the GAPDH-specific IgG antibody response by indirect ELISA (Song et al., 2017). Briefly, flat-bottomed 96-well plates (MarxiSorp, Nunc, Denmark) were coated overnight at 4°C with 100 μl solution per well of recombinant EaGAPDH or EmGAPDH (50 mg/ml) in 0.05 M carbonate buffer, pH 9.6. The plates were washed with 0.01 M PBS containing 0.05% Tween-20 (PBS-T) and blocked with 5% skim milk powder (SMP) in PBS-T for 2 h at 37°C. The plates were incubated for 2 h at 37°C with 100 μl of the serum samples diluted 1:50 in PBS-T with 1% SMP in duplicate. After three washes, the plates were incubated for 1 h at room temperature with 100 μl/well of HRP conjugated donkey anti-chicken IgG anti-body (Sigma–Aldrich) diluted 1:1000 in 2% SMP in PBS-T. Color development was carried out with 3, -3′, 5, 5′-tetramethylbenzidine (TMB) (Sigma-Aldrich), and the optical density at 450 nm (OD450) was determined with a microplate spectrophotometer. All serum samples were investigated by ELISA at the same time under the same conditions and were included on one plate.

### Protective Efficacy Evaluation of Coccidial Common Antigen GAPDH against Challenge with Three *Eimeria* Species

At 14 days of age, chickens were weighted and randomly divided into 17 groups of 30 chicks each (see **Table 3**). The schematic of vaccination was shown in **Figure 2B**. Experimental groups were intramuscularly vaccinated with 100 μg (0.5 μg/μl) of plasmids pVAX-EaGAPDH and pVAX-EmGAPDH in thigh, respectively. The vector control group was injected with 100 μg (0.5 μg/μl) of pVAX1, the challenged and unchallenged control groups were injected with sterile PBS buffer with the same administration route as the experimental groups. A booster vaccination was given 1 week later with the same amount of components as the primary vaccination. At 28 days of age, chickens were weighed individually and orally challenged with *E. tenella* (5 × 10^4^/bird), *E. acervulina* (10 × 10^4^ /bird), *E. maxima* (10 × 10^4^/bird), and mixed *Eimeria* species (5 × 10^4^ *E. tenella*/bird, 10 × 10^4^ *E. acervulina*/bird, 10 × 10^4^ *E. maxima*/bird) separately (Holdsworth et al., 2004), except the unchallenged control groups. Unchallenged control chickens were given PBS orally (see **Table 3**). Five to seven days, post challenge the chickens were weighed individually. Twenty chickens of each group were slaughtered for lesion score (Johnson and Reid, 1970). Additionally, the enteric contents of the chickens were collected separately and oocysts per gram of content (OPG) were counted using a McMaster chamber. The rest ten chickens were used to count the oocysts of the fecal samples during 5–10 days post-challenge. Efficacy of immunization was evaluated on the basis of survival rate, lesion score, body weight gain, oocyst decrease ratio and the ACI as described by Song et al. (2015a). The ACI is rated “good” when 180 or more, “moderate” when 160–179, and “poor” when below 160 (McManus et al., 1968).

**Table 3:** Protective efficacy of common antigen EaGAPDH against challenge with *E. tenella*, *E. acervulina*, *E. maxima*, and mixed oocysts of the three *Eimeria* species.

Group	Challenge with *Eimeria* spp.	Average body weightgain (g)	Relative body weight gain (%)	Mean lesionscores	Oocyst decrease ratio (%)	ACI
pVAX-EaGAPDH	*E. tenella*	48.28 ± 11.26^b^	79.54	0.53 ± 0.53^b^	85.98	169.24
pVAX-EmGAPDH	*E. tenella*	49.16 ± 12.23^b^	80.24	0.67 ± 0.72^b^	82.28	168.54
pVAX1 control	*E. tenella*	35.26 ± 9.89^c^	58.00	3.59 ± 0.51^c^	5.03	112.1
Challenged control	*E. tenella*	32.58 ± 10.29^c^	53.11	3.78 ± 0.67^c^	0.00	105.41
Unchallenged control^∗^	PBS	61.30 ± 9.29^a^	100	0 ± 0^a^	100	200

pVAX-EaGAPDH	*E. acervulina*	47.95 ± 8.47^bc^	79.57	1.35 ± 0.67^b^	58.59	165.07
pVAX-EmGAPDH	*E. acervulina*	52.11 ± 10.28^b^	87.07	1.46 ± 0.86^b^	55.21	171.47
pVAX1 control	*E. acervulina*	38.41 ± 10.93^c^	63.20	3.01 ± 0.81^c^	7.67	93.1
Challenged control	*E. acervulina*	37.38 ± 10.27^c^	62.37	3.26 ± 0.67^c^	0.00	89.77
Unchallenged control^∗^	PBS	61.30 ± 9.29^a^	100	0 ± 0^a^	100	200

pVAX-EaGAPDH	*E. maxima*	49.10 ± 9.26^b^	82.37	1.62 ± 0.75^bc^	44.14	165.17
pVAX-EmGAPDH	*E. maxima*	51.03 ± 12.45^b^	85.01	1.32 ± 0.81^b^	54.48	170.81
pVAX1 control	*E. maxima*	35.39 ± 11.52^c^	58.62	2.56 ± 0.71^c^	11.72	93.02
Challenged control	*E. maxima*	35.18 ± 12.21^c^	57.18	2.90 ± 0.67^c^	0.00	88.18
Unchallenged control^∗^	PBS	61.30 ± 9.29^a^	100	0 ± 0^a^	100	200

pVAX-EaGAPDH	Mixed oocysts	45.68 ± 9.76^b^	74.38	0.47 ± 0.61^b^	47.75	164.68
pVAX-EmGAPDH	Mixed oocysts	48.68 ± 10.21^b^	79.80	0.75 ± 0.54^b^	56.18	171.1
pVAX1 control	Mixed oocysts	35.10 ± 10.81^c^	57.57	3.58 ± 0.61^c^	–1.69	111.77
Challenged control	Mixed oocysts	33.08 ± 11.09^c^	53.77	3.70 ± 0.57^c^	0.00	106.77
Unchallenged control^∗^	PBS	61.30 ± 9.29^a^	100	0 ± 0^a^	100	200

*Significant difference (p < 0.05) between numbers with different letters. No significant difference (p > 0.05) between numbers with the same letter; ^∗^unchallenged control was shared among the groups challenged with different Eimeria species; mixed oocysts: mixed oocysts of E. tenella, E. acervuline, and E. maxima.*

### Statistical Analysis

One-way analysis of variance (ANOVA) with Duncan’s multiple range tests were used for the determination of statistical significance by using the SPSS statistical package (SPSS for Windows 16, SPSS Inc., Chicago, IL, USA). Differences among groups were tested and *p* < 0.05 was considered to indicate a significant difference.

## Results

### Cloning and Expression of *EaGAPDH* and *EmGAPDH*

**FIGURE 3:**
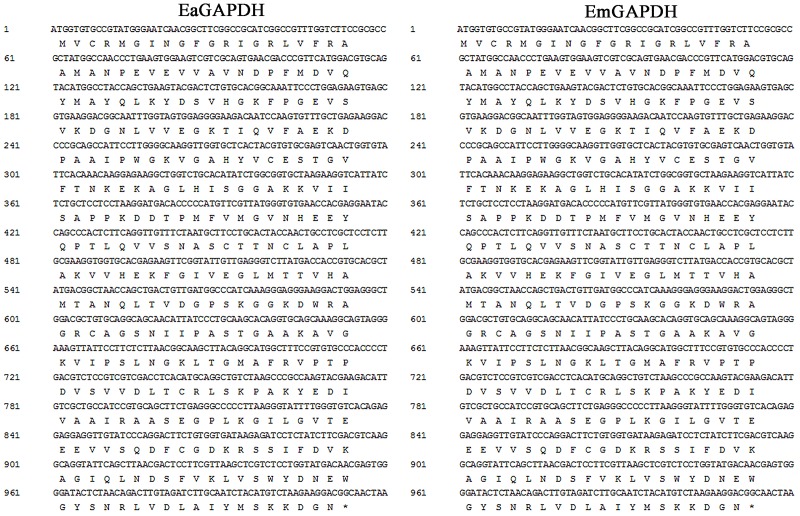
Open reading frames (ORFs) and deduced amino acid sequence of GAPDH.

**FIGURE 4:**
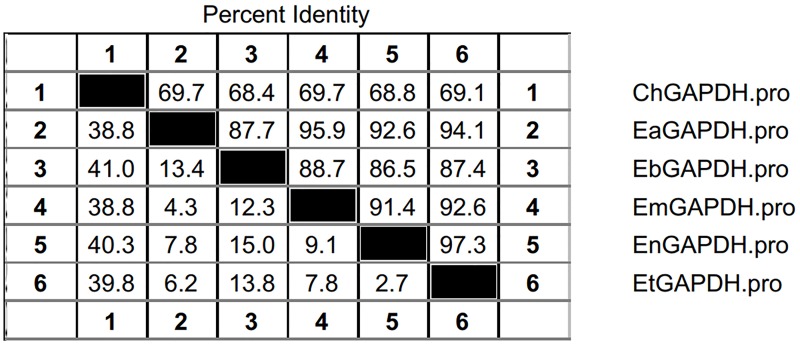
Amino acid similarities of GAPDH between *Eimeria acervulina*, *E. maxima*, *E. tenella*, *E. necatrix*, and *E. brunetti.* ChGAPDH = GAPDH of Chicken; EtGAPDH = GAPDH of *E. tenella*; EaGAPDH = GAPDH of *E. acervulina*; EmGAPDH = GAPDH of *E. maxima*; EnGAPDH = GAPDH of *E. necatrix*; EbGAPDH = GAPDH of *E. brunetti.*

**FIGURE 5:**
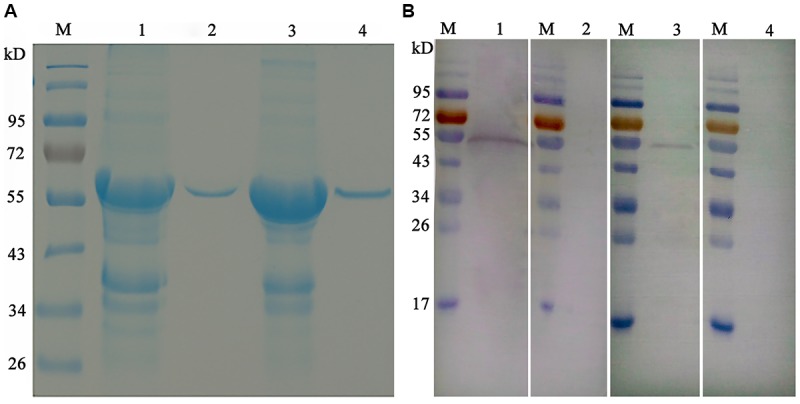
Purification and Western blot analysis of recombinant proteins of EaGAPDH and EmGAPDH with anti-*Eimeria* chicken sera. Lane M: standard protein molecular weight marker; **(A)** Purification of EaGAPDH and EmGAPDH; lane 1: Inclusion body extracted from pET32a-EaGAPDH transformed *E. coli*; lane 2: EaGAPDH purified through Ni_2_+-charged column chromatography; lane 3: Inclusion body extracted from pET32a-EmGAPDH transformed *E. coli*; lane 4: EmGAPDH purified through Ni_2_+-charged column chromatography; **(B)** Western blot analysis of recombinant proteins of EaGAPDH and EmGAPDH; lane1: EaGAPDH recognized by anti-*E. acervulina* chicken serum; lanes 2 and 4: EaGAPDH recognized by serum of uninfected chicken; lane 3: EmGAPDH recognized by anti-*E. maxima* chicken serum.

As shown in **Figure 3**, the open reading frames (ORFs) of *EaGAPDH* and *EmGAPDH* are composed 1020 nucleotides with predicted molecular weights of 37.4 kDa. BLAST analysis revealed that *EaGAPDH* and *EmGAPDH* shared similarities of 99% in nucleotides and amino acid sequences with the genes in NCBI (Gene ID: *EaGAPDH* 25337292; *EmGAPDH* 25268815), respectively. *GAPDH* shared similarities of more than 86% in amino acid among five species of chicken coccidia (**Figure 4**). The recombinant proteins of EaGAPDH and EmGAPDH were expressed as His_6_ tag fusion proteins. SDS–PAGE revealed prominent protein bands about 55.4 kDa (**Figure 5A**, lanes 1 and 3) which were consistent with molecular weight sum of fusion protein of pET-32a vector (18 kDa) and recombinant proteins of EaGAPDH or EmGAPDH (37.4 kDa). The purification of the recombinant proteins showed single band with molecular weight of 55.4 kDa by SDS–PAGE (**Figure 5A**, lanes 2 and 4). Western blot assay revealed specific band of 55.4 kDa (**Figure 5B**, lanes 1 and 3), indicating EaGAPDH and EmGAPDH recombinant proteins were recognized by with anti-*E. acervulina* and anti-*E. maxima* chicken sera, respectively.

### Identification of Plasmid of pVAX-EaGAPDH and pVAX-EmGAPDH

Plasmids pVAX-EaGAPDH and pVAX-EmGAPDH were generated using the eukaryotic expression vector pVAX1. The endonuclease cleavage and sequence analysis indicated that plasmids of pVAX-EaGAPDH and pVAX-EmGAPDH were constructed successfully.

### *GAPDH* was Transcribed and Expressed in the Injected Muscles of Chickens

**FIGURE 6:**
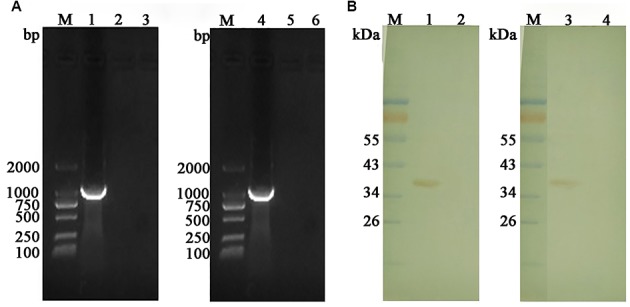
Transcription and expression detection of GAPDH by RT-PCR and Western blot in the injected muscles. **(A)** Transcription detection of GAPDH by RT-PCR; M: DL2000 DNA marker; lane 1: product of EaGAPDH from pVAX1-EaGAPDH injected muscle; lane 4: product of EmGAPDH from pVAX1-EmGAPDH injected muscle; lanes 2 and 5: pVAX1 injected control; lanes 3 and 6: non-injected control; **(B)** Expression detection of GAPDH by Western blot; M: Standard protein molecular weight marker; lane 1: protein band of 37.4 KDa recognized by anti-EaGAPDH rat serum from the muscles injected with pVAX-EaGAPDH; lane 2: non-injected muscle recognized with anti-EaGAPDH rat serum; lane 3: protein band of 37.4 KDa recognized by anti-EmGAPDH rat serum from the muscles injected with pVAX-EmGAPDH; lane 4: non-injected muscle recognized with anti-EmGAPDH rat serum.

Transcriptions of *GAPDH* gene in the injected muscles were detected using RT-PCR assay. With the specific primers of *EaGAPDH* and *EmGAPDH*, bands of approximately 1020 bp were detected from the muscle injected with pVAX-EaGAPDH and pVAX-EmGAPDH, respectively (**Figure 6A**, lanes 1 and 4). No specific DNA bands were detected in pVAX1 injected and non-injected control samples (**Figure 6A**, lanes 2 and 5; **Figure 6A**, lanes 3 and 6). This result indicated that *GAPDH* was transcribed in the injected muscles of chickens.

Expression the *GAPDH* genes in the injected muscle was detected by Western blot. As shown in **Figure 6B**, ant-EaGAPDH and ant-EmGAPDH sera reacted with protein band of approximately 37.4 kDa from the pVAX-EaGAPDH and pVAX-EmGAPDH injected muscles, respectively (**Figure 6B**, lanes 1 and 3). No specific band was detected in non-injected control samples (**Figure 6B**, lanes 2 and 4). This result indicated the eukaryotic recombinant plasmid pVAX-EaGAPDH and pVAX-EmGAPDH were expressed in the injected muscles of chickens.

### Administration with GAPDH Significantly Increased the Proportion of CD4^+^/CD3^+^ and CD8^+^/CD3^+^ T Lymphocytes Subpopulation in Chickens

**FIGURE 7:**
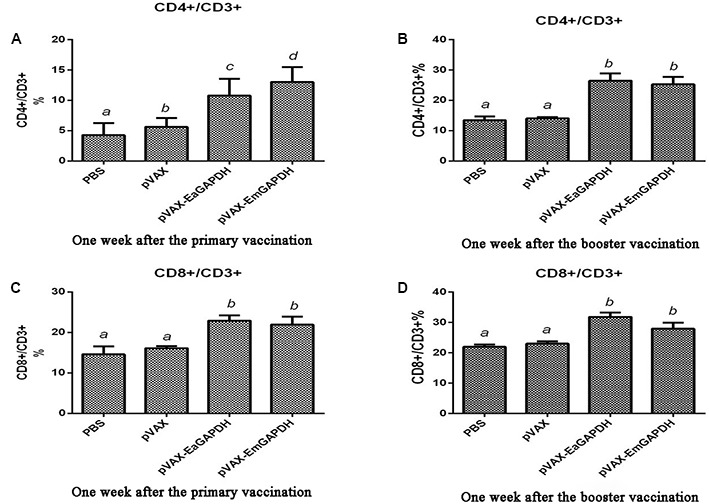
Changes of proportion of CD4+/CD3+ and CD8+/CD3+ T cells in spleens of the chickens vaccinated with pVAX-EaGAPDH and pVAX-EmGAPDH. **(A)** Proportion of CD4+/CD3+ T cells in spleens of the chickens 1 week after the primary vaccination; **(B)** proportion of CD4+/CD3+ T cells in spleens of the chickens 1 week after the booster vaccination; **(C)** proportion of CD8+/CD3+ T cells in spleens of the chickens 1 week after the primary vaccination; **(D)** proportion of CD8+/CD3+ T cells in spleens of the chickens 1 week after the booster vaccination; significant difference (*p* < 0.05) between numbers with different letters; non-significant difference (*p* > 0.05) between numbers with the same letter.

To determine the possible effects induced by GAPDH on T lymphocytes subpopulation, the proportions of CD4^+^/CD3^+^ and CD8^+^/CD3^+^ T cells in spleens of the vaccinated chickens were evaluated by flow cytometry (**Figure 7**). One week after the primary vaccination, administration with pVAX-EaGAPDH and pVAX-EmGAPDH significantly increased the proportion of CD4^+^/CD3^+^ and CD8^+^/CD3^+^ T cells compared to the pVAX1 and PBS control (*p* < 0.05). No significant difference was observed on the proportion of CD4^+^/CD3^+^ and CD8^+^/CD3^+^ T lymphocytes subpopulation between pVAX1 and PBS control group (*p* > 0.05). No significant difference was observed on the proportion of CD8^+^/CD3^+^ T lymphocyte subtype between pVAX-EaGAPDH and pVAX-EmGAPDH group (*p* > 0.05). However, pVAX-EmGAPDH significantly increased the proportion of CD4^+^/CD3^+^ T cells compared with pVAX-EaGAPDH group (*p* < 0.05). The similar result was observed one week after the booster vaccination, except that no significant difference was observed on the proportion of CD4^+^/CD3^+^ T cells between pVAX-EaGAPDH and pVAX-EmGAPDH group (*p* > 0.05).

### Administration with GAPDH Significantly Prompted the Cytokine Production in Chickens

**FIGURE 8:**
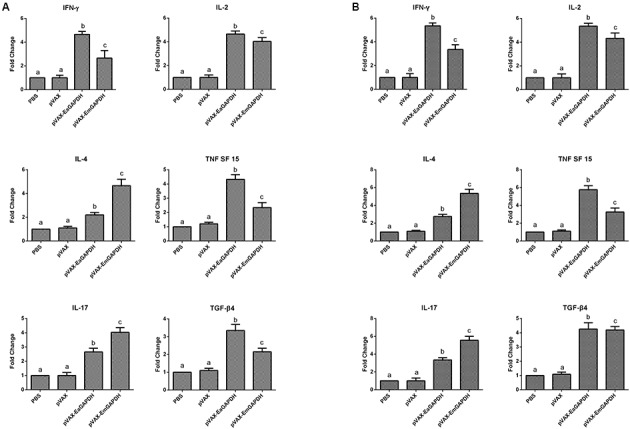
Changes of mRNA expression of cytokines in splenic lymphocytes following pVAX-EaGAPDH and pVAX-EmGAPDH vaccination. **(A)** One week after the primary vaccination; **(B)** One week after the booster vaccination; Significant difference (*p* < 0.05) between numbers with different letters; non-significant difference (*p* > 0.05) between numbers with the same letter.

To determine the possible effects induced by GAPDH on cytokine production, the transcription levels of IFN-γ, IL-2, IL-4 TNFSF15, IL-17, and TGF-β4 were determined by qPCR (**Figure 8**). One week after the primary vaccination, chickens immunized with recombinant plasmids pVAX-EaGAPDH and pVAX-EmGAPDH showed a significant switch in their cytokine profiles, with a significant increase in IFN-γ, IL-2, IL-4 TNFSF15, IL-17, and TGF-β4 compared with the pVAX1 and PBS control (*p* < 0.05). No significant difference was observed between pVAX1 and PBS control group (*p* > 0.05). The similar result was observed one week after the booster vaccination.

### Administration with GAPDH Significantly Induced IgG Antibody Response

**FIGURE 9:**
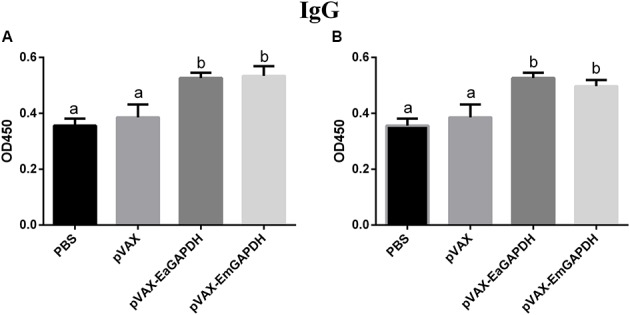
Serum IgG levels in chickens 1 week after the primary vaccination with pVAX-EaGAPDH and pVAX-EmGAPDH. **(A)** Serum IgG levels determined using ELISA coated with recombinant EaGAPDH; **(B)** Serum IgG levels determined using ELISA coated with recombinant EmGAPDH; significant difference (*p* < 0.05) between numbers with different letters; non-significant difference (*p* > 0.05) between numbers with the same letter.

**FIGURE 10:**
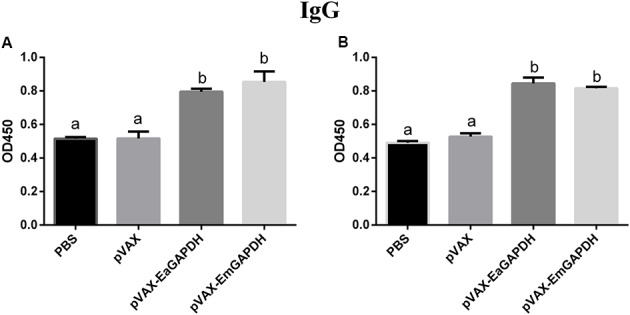
Serum IgG levels in chickens 1 week after the booster vaccination with pVAX-EaGAPDH and pVAX-EmGAPDH. **(A)** Serum IgG levels determined using ELISA coated with recombinant EaGAPDH; **(B)** Serum IgG levels determined using ELISA coated with recombinant EmGAPDH; significant difference (*p* < 0.05) between numbers with different letters; non-significant difference (*p* > 0.05) between numbers with the same letter.

To determine the possible effects induced by GAPDH on antibody response, levels of GAPDH-specific antibodies were determined by ELISA. One week after the primary vaccination, sera from chickens immunized with recombinant plasmids pVAX-EaGAPDH or pVAX-EmGAPDH showed significantly high level of IgG antibody (*p* < 0.05) compared to that of pVAX1 and PBS controls, whereas IgG antibody of chicken immunized with pVAX1 and PBS was not significantly induced (*p* > 0.05) (**Figure 9**). The similar result was observed one week after the booster vaccination (**Figure 10**).

### Administration with GAPDH Induced Effective Protection against Challenge with Three *Eimeria* Species

Protective efficacies of pVAX-EaGAPDH and pVAX-EmGAPDH were evaluated by challenge with *E. tenella*, *E. acervulina*, *E. maxima*, or mixed oocysts of the three *Eimeria* species, respectively. As shown in **Table 3**, body weight gains were significantly reduced in challenged and pVAX1 control group compared with the unchallenged control group (*p* < 0.05). Chickens vaccinated with pVAX-EaGAPDH and pVAX-EmGAPDH displayed significantly increased weight gains compared to chickens in challenged and pVAX1 control groups (*p* < 0.05). The oocyst outputs of pVAX-EaGAPDH and pVAX-EmGAPDH vaccinate chickens were significantly lower than those of challenged and pVAX1 control group (*p* < 0.05). Significant alleviations in enteric lesions were observed in pVAX-EaGAPDH and pVAX-EmGAPDH immunized chickens compared to those of challenged and pVAX1 control group (*p* < 0.05). Group of chickens immunized with recombinant plasmids pVAX-EaGAPDH and pVAX-EmGAPDH resulted in ACI more than 160 showing moderate protections against *E. tenella*, *E. acervulina*, *E. maxima*, and mixed infection of the three *Eimeria* species.

## Discussion

Clinical chicken coccidiosis is mostly caused by simultaneous infection of several *Eimeria* species, and host immunity against *Eimeria* is specie-specific (Carvalho et al., 2011; Ogedengbe et al., 2011; del Cacho et al., 2012). Therefore, it is a promise strategy to develop multivalent vaccine using effective common antigen among multiple *Eimeria* species to control the mixed infection of chicken coccidia. GAPDH is one of the immunogenic common antigens among *E. tenella*, *E. acervuline*, and *E. maxima* identified in our previous study. It is highly conserved among chicken coccidia (**Figure 1**). In this study, protective efficacy of the common antigen GAPDH was evaluated in form of DNA vaccine. Vaccination with pVAX-EaGAPDH and pVAX-EmGAPDH not only induced effective protection against infection of single *Eimeria* species (*E. tenella*, *E. acervuline*, and *E. maxima*), also the mixed infection of these species. Our study provided effective common antigen of GAPDH for the development of multivalent vaccine against co-infections of multiple *Eimeria* species in poultry farms.

DNA vaccines have been suggested as a promising alternative strategy against coccidiosis as they lack the disadvantages associated with chemo-prophylaxis and live vaccines (Ivory and Chadee, 2004; Blake and Tomley, 2014; Song et al., 2015c; Ahmad et al., 2016; Meunier et al., 2016). Therefore, we evaluated the immunogenicity and protective efficacy of common antigen GAPDH in form of DNA vaccine. The prerequisite for efficacy induction of DNA vaccine is that it could be expressed *in vivo*. Thus, we detected the expression of pVAX-EaGAPDH and pVAX-EmGAPDH from mRNA and protein level and found that GAPDH could be transcribed and expressed in the injected sites in chickens. These observations supported the protection against challenge with *Eimeria*.

The cell-mediated and humoral immune response induced by GAPDH in form of DNA vaccine was evaluated in this study. The proportions of CD4+ and CD8+ T lymphocytes were significantly increased by vaccination with pVAX-EaGAPDH and pVAX-EmGAPDH. Similarly, the transcriptions of six major cytokines (IFN-γ, IL-2, IL-4 TNFSF15, IL-17, and TGF-β4) were significantly increased by the vaccinations. The changes in T lymphocytes and cytokines indicated that a strong cell-mediated immune response was induced. Which is very important, because cell-mediated immune response plays a critical role in protective immunity against *Eimeria* infection (Dalloul and Lillehoj, 2006; Chapman, 2014). Although the role of humoral response during coccidiosis was debatable (Lillehoj and Trout, 1996; Yun et al., 2000), some researchers considered that humoral response could be of some relevance with protection. For example, the maternal anti gametocyte antibodies provided transmission-blocking immunity against *E. maxima* challenge in offspring chickens (Wallach et al., 1992; Dalloul and Lillehoj, 2006; Witcombe and Smith, 2014). In this study, administration with GAPDH significantly induced IgG antibody response. In short, GAPDH could induce significant cellular and humoral immune response as DNA vaccine which played a role in immune protection. Moreover, the native protein of GAPDH could react with the anti-*Eimeria* chicken serum and recombinant protein of GAPDH also could react with the anti-*Eimeria* chicken serum (Liu et al., 2017). These combined observations documented that the common antigen GAPDH possessed good immunogenicity.

In this study, although DNA vaccines of GAPDH provided moderate protection against *Eimeria*, there are some strategies could be considered to improve its protection (Ivory and Chadee, 2004). For example, immune responses induced by DNA vaccination can be enhanced by co-injection with recombinant cytokines or plasmids encoding these cytokines (Min et al., 2002; Song et al., 2015b). The efficacy of DNA vaccination also could be increased by optimization of vaccination procedure including route, dose, time of immunization and age of primary immunization of chickens (Song et al., 2009). Especially in the practice, some farmers prefer to give the broiler chickens a single immunization earlier than 2 weeks of age and deliver the vaccines with non-muscular injection route. Thus, the minimal age of immunization, the single immunization and the non-muscular delivery routes needs to be studied for the application of DNA vaccine of GAPDH in broiler chickens.

## Conclusion

The coccidal common antigen of GAPDH induced significant humoral and cellular response and protective efficacy against *E. tenella*, *E. acervulina*, *E. maxima*, and mixed infection of the three *Eimeria* species. These results documented GAPDH is a promising candidate common antigen for developing multivalent vaccine against the mixed infection of chicken coccidia clinically.

## Author Contributions

LT performed the animal experiments and drafted the manuscript; WL cloned the genes; XH prepared the antisera and revised the manuscript; DT performed the laboratory test; JL contributed to the qPCR analysis; LL contributed to the sample collecting; XY contributed to the Western blot analysis; RY, LX, and XL helped in the study design and analyzed the data; XS designed the study and critically revised the manuscript. All authors read and approved the final manuscript.

## Conflict of Interest Statement

The authors declare that the research was conducted in the absence of any commercial or financial relationships that could be construed as a potential conflict of interest.

## Footnotes

1 http://www.ncbi.nlm.nih.gov/BLAST/
